# Segmentation for pelvic malignancies in radiation oncology practice: a systematic review and meta-analysis protocol

**DOI:** 10.1186/s13643-026-03173-2

**Published:** 2026-04-23

**Authors:** Sidharth Satish Menon, Umesh Velu, Lavanya Gurram, Ganesh Paramasivam, Manjunath KN, Divya Susanna Patil, Vijay Shree Dhyani, Roshan David Jathanna, Shirley Lewis

**Affiliations:** 1 Department of Radiation Oncology, Kasturba Medical College, Manipal Academy of Higher Education, Manipal, India; 2 Department of Radiation Oncology, CancerCare Manitoba, Winnipeg, Manitoba R3E0V9 Canada; 3 Department of Cardiology, Kasturba Medical College, Manipal Academy of Higher Education, Manipal, India; 4 Manipal Institute of Technology, Manipal Academy of Higher Education, Manipal, India; 5 Department of Health Technology and Informatics, Prasanna School of Public Health, Manipal Academy of Higher Education, Manipal, India; 6 Kasturba Medical College, Manipal Academy of Higher Education, Manipal, India

**Keywords:** Autosegmentation, Dice coefficient score, Deep learning, Pelvic malignancy, Cancer, Convolutional neural network (CNN)

## Abstract

**Background:**

Deep learning (DL)-based artificial intelligence (AI) models, the fourth generation in autosegmentation, have been adopted both for commercial and research applications worldwide and have shown great promise as reliable and comprehensive resource strategies for radiotherapy workflows.

**Methods:**

A comprehensive search will be conducted on Medline (PubMed), Scopus, the Cochrane Library, EMBASE, and Web of Science between January 2004 and December 2025. We will conduct a title, abstract, and full-text screening of all studies as per the eligibility criteria. Two reviewers will be involved in screening studies, quality appraisal, and data extraction, and a third reviewer will be consulted to resolve conflicts. Based on data availability, the data will be synthesised via meta-analysis and narrative synthesis. The reporting will be performed using the Preferred Reporting Items for Systematic Reviews and Meta-Analyses (PRISMA) guidelines, ensuring the reliability and validity of the results.

**Discussion:**

The model’s performance will be assessed using various quantitative indices, qualitative tools, timesavings, and dosimetry. This review will enable us to determine the accuracy of autosegmentation for targets and various pelvic organs, boosting clinicians’ confidence in facilitating the clinical implementation of such tools in routine clinical practice.

**Systematic review registration:**

The protocol is prospectively registered on PROSPERO. The registration ID is CRD42024491066.

**Supplementary Information:**

The online version contains supplementary material available at 10.1186/s13643-026-03173-2.

## Background

Cancer has been a socioeconomic health problem since time immemorial and is among the three leading causes of death in those aged 30–69 years in over 177 countries [1]. There were approximately 20 million new cases and 9.7 million deaths due to cancer in 2022 [1]. Pelvic cancers, such as those of the colorectum, prostate, cervix uteri, and bladder, are among the top 10 leading cancers worldwide [1]. This global health concern requires equitable, comprehensive, and high-quality multimodal management involving diagnostic imaging, histopathology, and interventions such as surgery, chemotherapy, targeted therapy, and radiotherapy. It is estimated that nearly 50% of cancer patients will require one course of radiotherapy as a part of their therapy [2–4]. The availability of radiotherapy centres and machines is contingent on the national health framework and the socioeconomic status of nations. Currently, the density of radiotherapy machines per million people ranges between 0 and 0.4 in low- and middle-income countries (LMICs) [5]. The International Atomic Energy Agency (IAEA) recommends four radiotherapy units per million people. In LMICs, one radiotherapy unit serves 1–5 million people [6]. Such disparate access to healthcare, confounded by a high burden of cancer, results in a delay in the initiation of radiotherapy. This gap must be addressed by systematically recognising the current and future requirements and unmet needs. Consolidated efforts are required for quality radiotherapy, prioritising access at all levels of cancer care and addressing gaps in the quality and quantity of access to modern radiotherapy in LMICs [7].

The radiotherapy workflow requires human input every step of the way, the most critical being the segmentation and planning process. Variations in human knowledge, experience, and other factors in these steps have been shown to negatively impact the quality of care delivered and patient outcomes [8]. Artificial intelligence (AI) and its various functions, such as machine learning (ML) and deep learning (DL), have revolutionised the field of oncology care and radiation oncology alike. The most common applications of AI in radiotherapy are automatic segmentation, treatment planning, and synthetic CT generation [9].

Manual segmentation demands precise clinical and radiological experience. Traditionally, physicians perform manual segmentation, which is an elaborate and time-consuming task. It is highly physician-dependent and prone to interobserver error and bias [10]. Interobserver variation (IOV) can significantly impact the plan and, eventually, the clinical treatment and outcomes, as reported in multiple studies that included various sites, such as nasopharyngeal carcinoma, non-small cell lung cancer, and pancreatic cancer [11–13]. With the emergence of AI and its ensuing ML and DL models in medical image segmentation, autosegmentation has become an efficient and time-intensive tool for clinicians that may allow a productive workflow with improved dosimetry and possibly better clinical outcomes [14–17].

The earliest examples of autosegmentation techniques were limited by inferior computational power and were known as low-level segmentation approaches. They are clustered into four generations, the first of which is image intensity identification or aligning homogenous pixels by a preset criterion or region growing [18]. The second generation involved techniques such as k-means clustering, k-nearest neighbour, and Bayesian classifiers, which can classify and allocate voxels to designated clusters, allowing more accurate image segmentation [19–21]. The third-generation techniques were model-based and atlas/multiatlas-based segmentation, wherein manual segmentation is propagated via shape or appearance models or from libraries of reference images onto the desired imaging dataset [22–24]. The fourth generation incorporates conventional radiomics and filters before feature extraction and has been shown to be superior to the older iterations of autosegmentation applications. The subset of ML known as DL uses multiple designated neural layers, learns kernels/filters, and transforms the input image into the output. Through training, the convolutional layers in the network understand the initial filters once each specific edge, shape, and texture is exposed. Each layer will generate an activation map, all connected through the neurons, allowing the network to learn variations locally and globally [25, 26]. Several publications have reported on AI-based models for autosegmentation of target volumes and organs at risk (OARs) for pelvic cancers such as cervical, rectal, and prostate cancer [27–29]. The number of training datasets varies, and training is typically performed on computed tomography (CT) datasets. The model’s accuracy ranges from 70 to 95%, as per the Dice similarity coefficient (DSC), a popular quantitative scoring parameter. Few studies have reported on the impact of dosimetry and time savings [30–34]. There is significant variability among the studies about the number of datasets used for training and validation, the type of models, ground truth used, different imaging modalities, and different techniques which impacts their generalisability. The models trained on smaller tumours seen in developed nations may not be translatable for larger tumours prevalent in lower-middle-income countries.

### Rationale

Numerous autosegmentation applications are available, but implementing and integrating them into the clinical workflow remain a challenge. There is a need for performance assessment across multiple levels before implementation in clinical practice. The approaches for evaluating autosegmentation applications include geometric methods, dosimetry, timesaving metrics, and qualitative assessments [35]. These metrics are compared to the benchmark gold standard to determine the accuracy of segmentation [36]. The ground truth may be a single expert’s manual segmentation, a consensus segmentation, or a probabilistic estimate known as the simultaneous truth and performance level estimation (STAPLE) algorithm [37]. This systematic review helps shed light on the landscape of metrics used in the evaluation of autosegmentation algorithms and their accuracy in comparison to the various ground truths used in studies. Information on the varying quantitative and qualitative scores, dosimetric parameters, and time-related metrics of the autosegmentation models will help oncologists make informed decisions for the use of the models in trials and their integration into clinical practice.

## Methods/design

The protocol for this systematic review was developed following the Preferred Reporting Items for Systematic Review and Meta-Analysis Protocols (PRISMA-P) guidelines [38]. This information is provided in Additional file 1.

### Research question

Using the Population, Intervention, Comparison/Comparator, and Outcome(s) (PICO) framework for developing a research question to detail the importance of the research question, we propose the following research question [39].

What is the accuracy of autosegmentation compared to manual segmentation for target volumes and OARs in pelvic malignancies?

PICO framework for formulating the research question: as shown in Table 1.

**Table 1:** PICO framework

Population (P)	Pelvic cancers, for organs at risk and target volumes
Intervention (I)	Autosegmentation by models of various generations
Comparator (C)	Manual segmentation which is the gold standard
Outcome (O)	Performance of autosegmentation in terms of quantitative scores, qualitative measures such as subjective assessment, time saving, and dosimetry

### Primary objective

To study the performance of autosegmentation compared to manual segmentation, with the help of various indices.

### Secondary objectives

1. To review the DL models used for autosegmentation.
2. To review the indices and methods used to assess the accuracy of DL-based models.

The systematic review and meta-analysis will be conducted in accordance with PRISMA guidelines [40].

### Eligibility criteria

The eligibility criteria are provided in Table 2.

**Table 2:** Eligibility criteria

	Inclusion criteria	Exclusion criteria
Population (P)	Empiric research reporting on autosegmentation for pelvic malignancies like rectal cancer, anal canal cancer, prostate cancer, bladder cancer, cervical cancer, endometrial cancer, and vaginal or vulval cancer Empiric research reporting on autosegmentation from pelvic malignancies treated with neoadjuvant, adjuvant, or definitive radiation	Empiric research reporting on non-pelvic malignancies
Intervention (I)	Empiric research reporting on autosegmentation using AI/DL/ML methods	Empiric research reporting on autosegmentation using atlas-based or convolution methods
Comparator (C)	Empiric research comparing AI-generated autosegmentation with manual segmentation	Empiric research that have not evaluated autosegmentation models against manual segmentation
Outcome (O)	Empiric research reporting on efficacy of autosegmentation using any of the methods—Dice coefficient, Jaccard index, Hausdorff distance, average distance, etc.	Empiric research that have not evaluated performance of autosegmentation models
Study design	Empiric research in English language Studies in the form of randomised controlled trials (RCT), phase I, II trials, observational studies (retrospective and prospective), and population-based studies that report on autosegmentation	Research in progress, abstracts, case reports, dissertations, theses, books, book chapters, commentary, systematic reviews, meta-analysis, and guidelines
Time frame	January 2004 to December 2025	Empiric research performed before January 2004

#### Inclusion criteria

1. Empiric research reporting on autosegmentation using AI/DL/ML methods.
2. Empiric research comparing AI-generated auto-contours with manual contours.
3. Empiric research reporting on autosegmentation for pelvic malignancies like rectal cancer, anal canal cancer, prostate cancer, bladder cancer, cervical cancer, endometrial cancer, and vaginal or vulval cancer.
4. Empiric research reporting on autosegmentation from pelvic malignancies treated with neoadjuvant, adjuvant, or definitive radiation.
5. Empiric research reporting on autosegmentation from single or multi-centre studies.
6. Empiric research reporting on the efficacy of autosegmentation using any of the methods—Dice coefficient, Jaccard index, Hausdorff distance, average distance, etc.
7. The English language is only included.
8. Studies in the form of randomised controlled trials (RCTs), phase I and II trials, observational studies (retrospective and prospective), and population-based studies that report on autosegmentation.

#### Exclusion criteria

1. Research in progress, case reports, dissertations, theses, books, book chapters, commentary, systematic reviews, meta-analysis, and guidelines.
2. Empiric research reporting on autosegmentation using atlas-based or convolution methods.

### Information sources

The databases used for the literature search will be Medline (PubMed), Scopus, Web of Science, Cochrane Library, and EMBASE.

### Search strategy

This systematic review incorporates the MeSH terms related to ‘deep learning’, ‘pelvic malignancies’, and ‘artificial intelligence’, representing the concept of the studies, with the use of free text terms such as ‘autosegmentation’, ‘segmentation’, ‘cervical’, ‘rectal’, ‘bladder’, and ‘endometrial’. ‘Prostate’, ‘vaginal’, ‘vulval’, and ‘anal canal’ represent the various sites in the pelvis on which autosegmentation is being studied as an intervention. The search concepts captured in the form of MeSH terms and text words have been provided in Additional file 2. The preliminary search strategy employed on PubMed is provided in Additional file 3.

The Boolean operators ‘AND’ and ‘OR’ will be used in the string of searches in the respective databases. The ‘evidence-based manual for Peer Analysis of Electronic Search Strategies (PRESS) 2015 checklist for systematic searches’ will be used to report the search strategy systematically [41]. The screening will be limited to titles and abstracts of studies reported in English. All studies identified will be exported into data management software Covidence [42].

### Selection process

All citations from the database search will be uploaded to Covidence for deduplication and screening. We would begin with the removal of duplicates. The summated list of studies will be screened for duplicates, followed by screening for title and abstract by two independent reviewers against the stated eligibility criteria. The full texts of the relevant papers agreed upon by the authors will be procured and screened independently. A third reviewer will resolve the conflicts as per the eligibility criteria as and when necessary. The data obtained on the number of titles, abstracts included/excluded, the reasons for the same, and the number of full texts obtained will be depicted in the PRISMA 2020 flowchart [40].

### Data collection process

Data from the full texts will be extracted and filed into a preset data extraction sheet, which will be pilot tested on a few studies. Two independent reviewers will extract data from the full texts, and a third reviewer will intervene to resolve disagreements. An extraction sheet will summarise the details of the author, year of publication, aim of the study, participants/disease site, intervention/‘DL-based models’ implemented, details of training/validation/test sets used, time frame, imaging modality, evaluation metrics used, results, and other critical findings. Any missing data will be reported as ‘not available/not reported on’.

### Data items

Our goal is to compare autosegmentation to manual segmentation, and the following would be the data items:

1. ‘DL-based models’—The type of DL-based model used in the study, such as CNNs or variations of the same model, such as AlexNet, U-Net, and Res-Net. Other models such as generative adversarial network (GAN) and transformer-incorporated DL architectures.
2. Ground truth: The ground truth or manual contours used in each study will be captured such as manual contour by a single oncologist, peer-reviewed contours, consensus, or STAPLE based.
3. The primary tumour site cervix, endometrium, rectum, bladder, and prostate.
4. Imaging modality: The type of imaging modality as per routine standard of care for the respective cancer sites. Autosegmentation using computed tomography (CT), magnetic resonance imaging (MRI), or positron emission tomography (PETCT) planning scans.
5. Type of radiotherapy—External beam radiotherapy or brachytherapy.
6. Real-world clinical implementation of autosegmentation models—The type of implementation (pilot/prospective), the impact on clinical workflow in terms of throughput, quantitative metrics, dosimetry, and time saving in prospective real-world studies. Monitoring of autosegmentation models using quality assurance programs and capturing data on model drift and its potential impact on model performance.
7. Intent of radiotherapy—Curative intent, including radical or adjuvant radiotherapy and palliative intent radiotherapy.
8. Assessment tool:
  A. The quantitative indices include the Dice coefficient, the Jaccard index, and the Hausdorff index.
  B. Qualitative analysis—Likert scale, Turing test, and subjective grading tools.
  C. Time saving—Time taken for autosegmentation instead of the time taken for segmentation by a physician.
  D. Dosimetry—A dosimetric impact of autosegmentation compared with manual segmentation and to determine deviations in the doses received by organs or targets.
  E. Clinical outcomes like recurrence rates and survival rates—disease-free survival or overall survival with and without autosegmentation.
9. Datasets:
  A. Training set—The dataset used to train the model, where the model learns to recognise the desired structures or patterns.
  B. Test set—An independent dataset unseen during training used to assess the performance of the fully trained model, providing an unbiased evaluation of the model’s accuracy and effectiveness.
  C. Validation set—A separate dataset used to evaluate the model’s performance during training and to fine-tune the model’s parameters ensures that the model generalises well to unseen data.
  D. Number of datasets used for training, testing, and validation.

### Risk of bias assessment

Each included full text will be independently reviewed by two authors to whom the study will be blindly assigned. The included studies will be assessed using the Minimum Information about clinical artificial intelligence modelling (MI-CLAIM) checklist, designed for evaluating AI models in medical imaging [43]. Discrepancies will be resolved by discussion with a third reviewer. Studies with unclear and/or disparate risk of bias owing to multiple domains with high and low scores will be reported transparently. A funnel plot and Egger’s publication bias test will be generated to assess publication bias using RevMan software [44]. We will assess potential for optimism bias during critical appraisal of studies by assessing representativeness, reporting of negative results, and justification of performance metrics.

### Effect measures

As the quantitative scores are summary statistics, the mean scores of the quantitative indices will be reported, such as that of the Dice coefficient score and Jaccard index, and their standard deviation. A forest plot will also be generated to represent the accuracy using the mean difference (continuous outcome) of indices of segmentation of target volumes and/or organs at risk, dosimetry, time saving, and risk difference for qualitative scores (dichotomous outcome).

### Data synthesis

All studies that have compared autosegmentation models with manual segmentation will be included. All the characteristics of the studies, such as the author, year of publication, the AI model used, size of training/validation/test datasets, tumour site, imaging modality CT/MRI and radiation technique (External beam radiotherapy (EBRT) or brachytherapy), the ground truth used, and the intent of radiotherapy, will be tabulated and reported narratively. A random effects meta-analysis will be performed and illustrated using forest plots. A forest plot will depict the model’s accuracy based on the scores of various quantitative indices based on each tumour site, imaging modality CT/MRI, and radiation technique (EBRT or brachytherapy). Pearson’s correlation test will be used to describe the correlation between the score of the quantitative index and dosimetric impact and, subsequently, to help indicate a clinically meaningful outcome. The *I*^2^ test and a random effects model will be used to assess heterogeneity among the studies. The subgroup analysis, along with meta-regression, will be performed for type of model, type of ground truth, and number of datasets used for each site.

### Certainty assessment

We will use the Grading of Recommendations Assessment, Development and Evaluation (GRADE) approach by evaluating the risk of bias, inconsistency, imprecision, indirectness, and publication bias [45]. This gives an overall rating of certainty of evidence and the likelihood of the pooled effect of the desired outcomes, i.e. desirable scores of quantitative indices, qualitative measures, time saving, and dosimetry.

### Reporting

This systematic review will be performed and reported in accordance with PRISMA guidelines [40]. Missing data will be compiled by contacting the corresponding authors of the respective studies.

## Discussion

AI and ML applications have permeated every step of the radiotherapy workflow. Multiple original research ventures from LMICs involving the optimisation of radiotherapy planning, treatment delivery, and quality implementation have been developed in the past few years, demonstrating improved efficiency in executing repetitive tasks and aiding decision-making [46]. Autosegmentation aims to achieve reduced IOV and improved dosimetry in minimal segmentation time. At present, there are no standardised guidelines for evaluating autosegmentation models, or recommendations for the ground truth and the number of training or validation datasets that would be appropriate for a medical image segmentation study. Although time-saving and qualitative scoring systems have shown good concordance with clinically meaningful outcomes [35], there is a considerable lack of correlation between the quantitative scores of overlap metrics such as the Dice coefficient, dosimetric analysis, and clinically meaningful outcomes.

This systematic review would be one of the first to evaluate AI based autosegmentation models against manual segmentation for various pelvic cancers. We will systematically address the benefits and deficiencies of parameters of each domain, clinical relevance, in contrast to manual segmentation, and obtain a meta-analysis and thus produce a comprehensive profile from the pooled effects of outcomes of the autosegmentation models. This will help inform decision-making, the development of quality implementation programs, and the clinical application of autosegmentation models.

## Supplementary Information

Supplementary Material 1

Supplementary Material 2

Supplementary Material 3

## Data Availability

The data supporting the protocol in this article are included within the article and its additional files.

## Abbreviations

ML: Machine learning
DL: Deep learning
PRISMA: Preferred reporting items for Systematic Reviews and Meta-Analyses
CNN: Convolutional neural network
AI: Artificial intelligence
IOV: Interobserver variation
CT: Computed tomography
STAPLE: Simultaneous truth and performance level estimation
RCT: Randomised clinical trial
PRESS: Peer Analysis of Electronic Search Strategies
GAN: Generative adversarial network
GRADE: Grading of Recommendations Assessment, Development and Evaluation
LMIC: Low- and Middle-Income Countries
IAEA: International Atomic Energy Agency
PRISMA-P: Preferred Reporting Items for Systematic Review and Meta-Analysis Protocols
PICO: Population Intervention Comparator outcome
MRI: Magnetic resonance imaging
PET: Positron emission tomography
EBRT: External beam radiation
