# Supplementary material for: Segmentation for pelvic malignancies in radiation oncology practice: a systematic review and meta-analysis protocol
Source: Syst Rev. 2026 Apr 23;15:187. doi: 10.1186/s13643-026-03173-2 (PMC13237914; doi:10.1186/s13643-026-03173-2)
Supplement: Supplementary file 2 — Supplementary Material 2 [file 13643_2026_3173_MOESM2_ESM.docx]

**Database search strategy**

**PubMed**

|  | Key Concept 1 | Key Concept 2 | Key Concept 3 | Key Concept 4 |
| --- | --- | --- | --- | --- |
| MeSH | “Artificial Intelligence” OR “Machine Learning” OR “Deep Learning” | “Pelvic Neoplasms” OR “Uterine Cervical Neoplasms” OR “Rectal Neoplasms” OR “Urinary Bladder Neoplasms” OR “Endometrial Neoplasms” OR “Prostatic Neoplasms” OR “Vaginal Neoplasms” OR “Vulval Neoplasms” | “Organs at Risk”  No MeSH terms available for “Auto-segmentation” OR “Auto-contouring” OR “Contouring” | “Radiotherapy”  OR “Brachytherapy” |
| Free text words | “DL” OR “AI” OR “CNN” OR “Convolutional Neural Networks” | “Pelvic cancers” OR “Cervix cancer” OR “Rectal cancer” OR “Endometrial Cancer” OR “Prostate Cancer” OR “Vaginal cancer” OR “Vulval cancer” OR “Anal canal cancer” | “Auto-segmentation” OR “Autosegmentation” OR “Auto-contouring” OR “Contouring” OR “Target Volumes” OR “Automatic” | “Radiation Therapy”  OR “EBRT” |
