# Supplementary material for: Segmentation for pelvic malignancies in radiation oncology practice: a systematic review and meta-analysis protocol
Source: Syst Rev. 2026 Apr 23;15:187. doi: 10.1186/s13643-026-03173-2 (PMC13237914; doi:10.1186/s13643-026-03173-2)
Supplement: Supplementary file 3 — Supplementary Material 3 [file 13643_2026_3173_MOESM3_ESM.docx]

**Search Strategy**

| Key concept 1 | "DL"[Text Word] OR "AI"[Text Word] OR "CNN"[Text Word] OR "convolutional neural networks"[Text Word] OR "artificial intelligence"[MeSH Terms] OR "machine learning"[MeSH Terms] OR "deep learning"[MeSH Terms] |
| --- | --- |
| Key concept 2 | "pelvic cancers"[Text Word] OR "cervical cancer"[Text Word] OR "rectal cancer"[Text Word] OR "endometrial cancer"[Text Word] OR "prostate cancer"[Text Word] OR "vaginal cancer"[Text Word] OR "vulval cancer"[Text Word] OR "anal canal cancer"[Text Word] OR "bladder cancer"[Text Word] OR "pelvic neoplasms"[MeSH Terms] OR "uterine cervical neoplasms"[MeSH Terms] OR "rectal neoplasms"[MeSH Terms] OR "urinary bladder neoplasms"[MeSH Terms] OR "endometrial neoplasms"[MeSH Terms] OR "prostatic neoplasms"[MeSH Terms] OR "vaginal neoplasms"[MeSH Terms] OR (("vulva"[MeSH Terms] OR "vulva"[All Fields] OR "Vulval"[All Fields]) AND "neoplasms"[MeSH Terms]) |
| Key concept 3 | "Auto-segmentation"[Text Word] OR "Autosegmentation"[Text Word] OR "Auto-contouring"[Text Word] OR "Contouring"[Text Word] OR "target volumes"[Text Word] OR "target volumes"[Text Word] OR "Automatic"[Text Word] OR "organs at risk"[MeSH Terms] |
| Key concept 4 | "radiation therapy"[Text Word] OR "EBRT"[Text Word] OR "radiotherapy"[MeSH Terms] OR "brachytherapy"[MeSH Terms] |
| Collated Search = Key concept 1 + Key Concept 2 + Key Concept 3 + Key Concept 4 | ("radiation therapy"[Text Word] OR "EBRT"[Text Word] OR "radiotherapy"[MeSH Terms] OR "brachytherapy"[MeSH Terms]) AND ("Auto-segmentation"[Text Word] OR "Autosegmentation"[Text Word] OR "Auto-contouring"[Text Word] OR "Contouring"[Text Word] OR "target volumes"[Text Word] OR "target volumes"[Text Word] OR "Automatic"[Text Word] OR "organs at risk"[MeSH Terms]) AND ("pelvic cancers"[Text Word] OR "cervical cancer"[Text Word] OR "rectal cancer"[Text Word] OR "endometrial cancer"[Text Word] OR "prostate cancer"[Text Word] OR "vaginal cancer"[Text Word] OR "vulval cancer"[Text Word] OR "anal canal cancer"[Text Word] OR "bladder cancer"[Text Word] OR "pelvic neoplasms"[MeSH Terms] OR "uterine cervical neoplasms"[MeSH Terms] OR "rectal neoplasms"[MeSH Terms] OR "urinary bladder neoplasms"[MeSH Terms] OR "endometrial neoplasms"[MeSH Terms] OR "prostatic neoplasms"[MeSH Terms] OR "vaginal neoplasms"[MeSH Terms] OR (("vulva"[MeSH Terms] OR "vulva"[All Fields] OR "Vulval"[All Fields]) AND "neoplasms"[MeSH Terms])) AND ("DL"[Text Word] OR "AI"[Text Word] OR "CNN"[Text Word] OR "convolutional neural networks"[Text Word] OR "artificial intelligence"[MeSH Terms] OR "machine learning"[MeSH Terms] OR "deep learning"[MeSH Terms]) |
